# The Open Service Signal in Space Navigation Data Comparison of the Global Positioning System and the BeiDou Navigation Satellite System

**DOI:** 10.3390/s140815182

**Published:** 2014-08-19

**Authors:** Shau-Shiun Jan, An-Lin Tao

**Affiliations:** Institute of Aeronautics and Astronautics, National Cheng Kung University, Tainan 70101, Taiwan; E-Mail: taoanlin@gmail.com

**Keywords:** Global Navigation Satellite System (GNSS), BeiDou Navigation Satellite System (BDS), Global Positioning System (GPS), navigation data, ephemeris, almanac

## Abstract

More and more Global Navigation Satellite Systems (GNSSs) have been developed and are in operation. Before integrating information on various GNSSs, the differences between the various systems must be studied first. This research focuses on analyzing the navigation data differences between the Chinese BeiDou Navigation Satellite System (BDS) and the United States' Global Positioning System (GPS). In addition to explaining the impact caused by these two different coordinate and time systems, this research uses an actual open service signal in space (SIS) for both GPS and BDS to analyze their current system performance. Five data quality analysis (DQA) mechanisms are proposed in this research to validate both systems' SIS navigation data. These five DQAs evaluate the differences in ephemeris and almanac messages from both systems for stability and accuracy. After all of the DQAs, the different issues related to GPS and BDS satellite information are presented. Finally, based on these DQA results, this research provides suggested resolutions for the combined use of GPS and BDS for navigation and guidance.

## 1. Introduction

Due to the fact that the Global Navigation Satellite System (GNSS) has the ability to provide position, velocity and time information to all users near the Earth's surface, many countries have focused on the development of their own GNSSs for strategic considerations [1]. Currently, the United States' Global Positioning System (GPS) and the Russian Global Navigation Satellite System (GLONASS) are already providing services and are available to civilian users [2,3].

**Figure 1.**
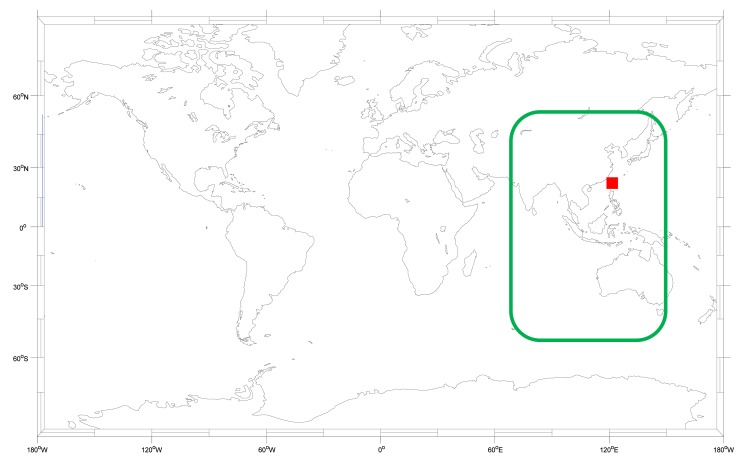
BDS service area.

Recently, the Chinese BeiDou Navigation Satellite System (BDS) has been developing rapidly and now meets the open service requirements for civilian users [4]. The service area of the current BDS is 55°S∼55°N, 70°E∼150°E, as shown in Figure 1. As a user in Taiwan, it is important to know the benefit of applying the BDS because we can receive the BDS satellite signals all day. This research is based on actual BDS signal in space (SIS) data which is used to analyze the differences between the BDS and the GPS. Moreover, this research also provides suggestions to users who want to combine both systems for positioning.

Compared with the current GPS constellation, which consists of 31 medium earth orbit (MEO) satellites, the BDS constellation had five Geostationary Earth Orbit (GEO) satellites, five Inclined Geosynchronous Orbit (IGSO) satellites, and four MEO satellites as of May of 2014. GPS does not have any GEO and IGSO satellites in the constellation. However, the two system's MEO satellites are not at the same orbit altitude. The orbit altitude of the BDS MEO satellites are 21,528 km, and the GPS satellites are operating at an altitude of 20,200 km. The GPS satellites appear at the same place at almost the same time because the periods for GPS satellites are about 12 h. Due to the fact that the orbit altitude of BDS MEO satellites is higher than that of the GPS satellites, the periods of the BDS MEO satellites are almost 13 h. As a result, the BDS satellite geometry will change for a fixed user at the same time every day.

Under the premise of different constellations, Table 1 illustrates the main differences between the BDS and GPS. The BDS coordinate system is the China Geodetic Coordinate System 2000 (CGCS2000), and the GPS coordinate system is the World Geodetic System 1984 (WGS84). Both of the coordinate systems are based on the idea of Earth-centered Earth-fixed (ECEF) coordinates, but the definition of the ellipsoid parameters is different. However, the maxima difference in the latitude and longitude between the WGS84 and CGCS2000 is less than 1.1 × 10^−3^ m [5]. The slight difference between the two coordinate systems will be ignored in this research. The BDS uses BeiDou navigation satellite system Time (BDT) for its time system, which started calculating at 00:00:00 on 1 January 2006 in Coordinated Universal Time (UTC). The GPS time (GPST) started at UTC at 00:00:00 on January 6th, 1980. The time difference between the BDT and GPST is about 14 s. Finally, the BDS signal's multiplexing mode is code division multiple access (CDMA), which is the same as that of the GPS [6]. Finally, this research analyzes the single frequency signal for a user for which the frequency of the BDS B1I signal is 1561.098 MHz, and the GPS L1 signal is 1575.42 MHz. The time system is based on the GPST to analyze the results for both systems (*i.e.*, GPST = BDT + 14).

Under ideal conditions, all the differences between the different GNSS constellations could be determined after the coordinate and time conversion calculations. In reality, however, there are many other issues need to be resolved when a user wants to integrate different GNSS signals. For example, in order to integrate the different GNSSs for a centimeter accuracy positioning by the conventional double difference method, the inter-system biases should be determined first [7]. The inter-system biases between different GNSSs affect the resolution of integer ambiguity when a user attempts to combine the measurements from several GNSS constellations. The study of different combinations of the GNSS differential inter-system biases is presented in [8]. In addition, because the special design of the BDS constellation includes GEO constellation, IGSO constellation and MEO constellation satellites, the BDS has inter-satellite-type biases between the different constellations which are described in [9].

Besides these inter-system biases and inter-satellite-type biases, it is of practical interest to investigate the SIS performance for GPS and BDS. The objective of the work presented here is to evaluate the differences between the two kinds of navigation data from GPS and BDS. GPS and BDS broadcast two kinds of satellite positioning information in the navigation data, almanac and ephemeris. In this research, both the ephemeris and almanac data will be verified for both systems. This research utilizes the GPS as a standard to compare the SIS data quality with the BDS. Five analysis methods will be presented to verify the consistency of the ephemeris and almanac data for both systems. The five data quality analyses (DQAs) discussed are as follows:

(1). Satellite position difference when ephemeris updates
(2). Satellite clock correction difference when ephemeris updates
(3). Ephemeris applicable period
(4). Satellite position difference between almanac and ephemeris
(5). Almanac applicable period

The SIS signal used in this paper is from 10 July to 13 August 2013. Both the GPS and BDS signals are recorded at the same time in the same place. The receiver is the NovAtel FlexPak 6 (NovAtel Inc., Calgary, AB, Canada), and the antenna is the NovAtel 703 GGG. The user is located at the National Cheng Kung University of Taiwan, which is also marked in Figure 1 as a red square. Each test method will be described in detail in Sections 2–6. Finally we present the conclusions and suggestions for combining GPS and BDS data.

## 2. Data Quality Analysis 1 (DQA1): Satellite Position Difference When Ephemeris Updates

**Figure 2.**
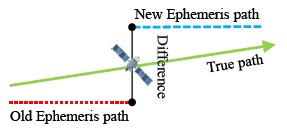
Schematic diagram of DQA1.

Ephemeris data is more precise than almanac data. Each satellite broadcasts its own ephemeris data. Before user positioning, ephemeris data for each satellite in view must be downloaded first. The information in the ephemeris data helps users to calculate the precise location, inclination and size of the satellite orbit. After that, the user can use the time information to calculate the satellite position on this orbit. However, the satellite position calculated from ephemeris data is not the true location of the satellite [10]. Due to the fact that an elliptical orbit cannot reflect the real dynamics of the satellite, satellite positions calculated from ephemeris data still have some errors. When one ephemeris updates its own satellite position, the new orbit parameters will create a new satellite orbit. If the satellite moves in a stable manner on its expected orbit, and the orbit parameters in the ephemeris data are provided correctly, the satellite position difference between the new and original ephemeris data will be under a certain value. As shown in Figure 2, there still is a slight difference because the error between the true location and different orbit *i.e.*, the new and old ephemeris orbit is not the same.

In this section, we use the true GPS and BDS SIS to acquire the ephemeris data and verify the satellite position difference calculated from each new and original ephemeris when the ephemeris updates. Before calculating the positioning result for each ephemeris data, the ephemeris is classified. The flag in Table 2 presents the state of each ephemeris flag. Flag 0 means the satellite did not have any ephemeris before. This satellite is a new incoming satellite, and it does not have any old ephemeris to provide a comparison. Flag 1 means this satellite already has an old ephemeris and that it is currently broadcasting a new ephemeris. Generally, the ephemeris data updates for a certain period. In the case of the GPS, the ephemeris updates every two hours, and in the case of the BDS, the ephemeris updates every hour. If one satellite is influenced and deviates from the original orbit, the ephemeris will update more quickly than its specified period. Flag 2 mark the ephemeris data for which the update time is over the predetermined time (GPS: 2 h, BDS: 1 h), and this flag also marks an unhealthy satellite. If one ephemeris flag is 2, this satellite ephemeris will be removed, and the next ephemeris will be waited for, which will be marked as flag 0. In this section, we only analyze cases of flag 1 ephemeris. The results are presented for each ephemeris update under normal conditions.

Another important quality index to assess the open service positioning accuracy of a satellite navigation system is the user range accuracy (URA). The URA definition is given in both the GPS interface control document (ICD) [2] and BDS ICD [4], and URA is a parameter in the ephemeris to provide a conservative RMS estimate of the user range error (URE) in the associated navigation data for the transmitting satellite. URA includes all errors for which the Space and Control Segments are responsible. That is, the URA presents the statistical result for the satellite ephemeris and clock errors, and it does not include the errors after the signal transmitted from the satellite (*i.e.*, the ionospheric, tropospheric and receiver errors). The comparison of the broadcasted URA values and the corresponding DQA1 results is given at the end of this section.

**Figure 3.**
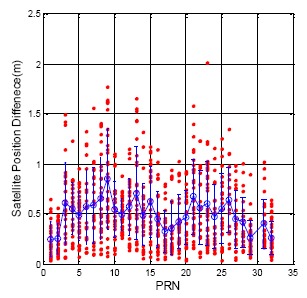
GPS DQA1: Satellite position differences between new and original ephemeris.

The calculation of the satellite position follows the GPS ICD and the BDS ICD. Figure 3 demonstrates the GPS satellite position difference for each satellite when the ephemeris updates (flag = 1). The analysis results are calculated for one week from 15 July to 22 July 2013. The horizontal axis presents each satellite number, and the vertical axis shows the position difference value. The red point in Figure 3 demonstrates the position difference for every satellite. The blue circle and blue bar represent the mean and the standard deviation value for each satellite, respectively. The Maxima of the update difference is 2.01 m. The total satellite mean value is 0.50 m, and the standard deviation is 0.30 m.

**Figure 4.**
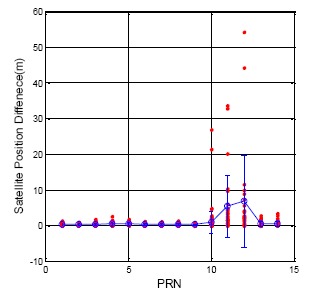
BDS DQA1: Satellite position difference between new and original ephemeris.

In order to make a comparison with the GPS, the ephemeris update results for the BDS are shown in Figure 4, and the statistical results are detailed in Table 3 for each satellite. In general, the BDS satellite position update difference is smaller than that of the GPS because the update rate of the BDS satellites is faster than that of the GPS satellites. Each ephemeris data will update within one hour for the BDS satellite. The GEO satellites exhibit the best satellite positioning results regardless whether or not the mean value and the standard deviation are generally better than those of the GPS. However, the IGSO (PRN 10) and MEO (PRN 11 and 12) exhibit dramatic differences during a normal ephemeris update. The maximum of the ephemeris update difference is 54.278 m. This sudden change in satellite position will lead to an increase in user positioning error. This information illustrates that the orbit prediction for the BDS is not as stable as that of the GPS for the BDS IGSO and MEO satellites.

**Figure 5.**
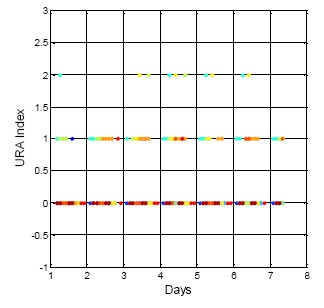
The broadcasted GPS URA index values for one week.

**Figure 6.**
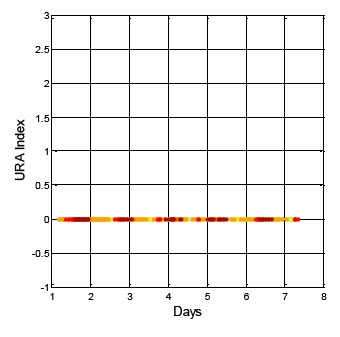
The broadcasted BDS URA index values for one week.

After analyzing the URA index values in the ephemeris for the same week (*i.e.*, 15 July to 22 July 2013) the URA index values show different results than that of DQA1. The GPS result is shown in Figure 5. The vertical axis indicates the URA index value, and the horizontal axis is time. The different colors in the figure are for the different satellite results. For GPS, the URA index values vary from 0 to 2, and the corresponding URA values are from 0.0 to 4.85 m (1σ) [2]. In general, the broadcasted GPS URA index values are stable and have few variations. On the other hand, the broadcasted BDS URA index values are shown in Figure 6. In the collected dataset for this specific week, the BDS URA index values are very stable at 0, that is, three constellations (GEO, IGSO and MEO) of BDS URA values are all within range of 0.0 to 2.40 m [4]. Based on the URA results, BDS satellites could provide stable positioning service to users with sufficient accuracy. Though the URA values are one-sigma (1σ) statistical results, the objective of the work presented here is to show that there are some rare irregularities in the broadcasted BDS ephemeris and almanac data that might be potential threat to the positioning services with more stringent requirements, for instance, the performance based navigation (PBN) for aviation. If there are sudden changes in satellite position and both the new and original ephemerides are healthy, and the update period between the two ephemerides is within one hour and the URA index values are the same, then there would be no way to confirm the consistency of the ephemeris data, unless the GNSS receiver uses the DQA1 test proposed in this research to verify each satellite position and remove the large change in the satellite position due to the new ephemeris update. Moreover, if users want to improve positioning accuracy under this circumstance, a precise orbit determination is needed as suggested in [11].

## 3. Data Quality Analysis 2 (DQA2): Satellite Clock Correction Difference When Ephemeris Updates

**Figure 7.**
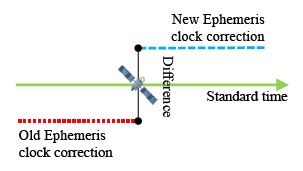
Schematic diagram of DQA2.

The satellite clock correction which calculates from the ephemeris must also be analyzed. Figure 7 shows that different ephemeris will broadcast different clock corrections for one satellite. Equation (1) presents the calculation of the satellite clock correction. The a_f0_, a_f1_ and a_f2_ are the polynomial coefficients given in the ephemeris data; t-t_oc_ presents the time transmit from the satellite to the user. The Δt_r_ is the relativistic correction term shown in Equation (2). The F, e and A are also given in the ephemeris data. The calculation of E_k_ is defined in the ICD [2,4]. The coefficient of the orbit parameters are not the same value due to the fact that the coordination systems of GPS and BDS are different. However, both systems use the same equation to calculate the satellite clock correction.

(1) $$\Delta {\text{t}}_{\text{sv}}={\text{a}}_{\text{f}0}+{\text{a}}_{\text{f1}}\left(\text{t}-{\text{t}}_{\text{oc}}\right)+{\text{a}}_{\text{f2}}{\left(\text{t}-{\text{t}}_{\text{oc}}\right)}^{\text{2}}+\Delta {\text{t}}_{\text{r}}$$

(2) $$\Delta {\text{t}}_{\text{r}}=\text{Fe}{\left(\text{A}\right)}^{\text{1}/\text{2}}{\text{sinE}}_{\text{k}}$$

**Figure 8.**
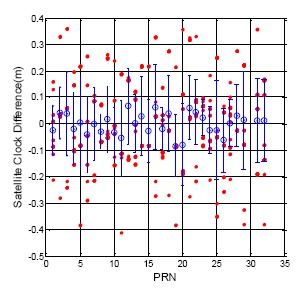
GPS DQA2: Satellite clock correction difference between new and original ephemeris.

**Figure 9.**
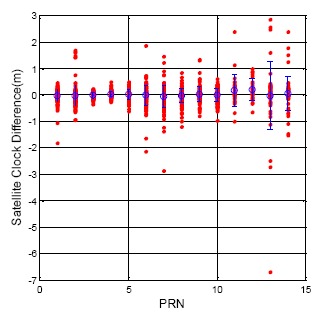
BDS DQA2: Satellite clock correction difference between new and original ephemeris.

Figures 8 and 9 show the clock correction difference as calculated from the different ephemeris data for the GPS and BDS, respectively. For all of the GPS satellites, the clock correction difference is under 0.5 m, and the standard deviation for each satellite is no more than 0.2 m. On the other hand, all the BDS satellite clock correction exhibit unstable changes when the ephemeris updates. The statistical results for all of the BDS satellite are shown in Table 4. The maximum clock correction difference for all BDS satellites is over 6 m, and the maximum value for each constellation is over one meter. This sudden change in the satellite clock correction will also influence user positioning when a BDS user receives a new ephemeris. This information shows the time synchronization for the BDS satellite still needs to be improved.

If a BDS user wants to reduce satellite clock error, the user can followed the Wide Area Differential Global Navigation Satellite System (WADGNSS) to estimate a more accurate clock correction [12].

## 4. Data Quality Analysis 3 (DQA3): Applicable Period for Ephemeris

**Figure 10.**
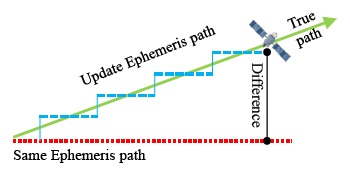
Schematic diagram of DQA3.

According to the official description, the GPS satellite updates its new ephemeris data every two hours and the BDS satellite updates every hour. Because of the weakness of ephemeris, the use periods are less than those for the almanac data. The ephemeris can be used for only a few hours. If one satellite dose not receives a new ephemeris, the original ephemeris should be removed after a certain time, or the expired ephemeris data will generate the wrong satellite position and clock correction for the user. In this section, the same ephemeris is used for six hours in order to compare the satellite positioning results with the updated ephemeris, as shown in Figure 10.

**Figure 11.**
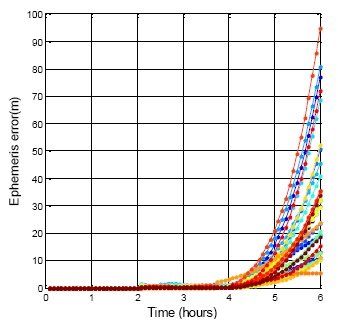
GPS DQA3: The same ephemeris used for 6 h.

For one user, each satellite is in receivable space at different times. The first requirement is to select the period for each satellite SIS data that continues for six hours. As a result, all the GPS and BDS satellites can be received for over six hours. Figure 11 presents all the GPS satellites using their own ephemeris for six hours. The different colors stand for different satellites. The updated ephemeris is used to calculate the true location of each satellite. In Figure 11, the slight jump at the two hours point is due to the new ephemeris difference as discussed in Section 2.1.1. However, after four hours, every GPS satellite position difference is dramatically increased. The maximum difference after six hours is 94.9 m, and the minimum is less than 10 m. As a result, if the GPS ephemeris does not update when it need to, the available period of the ephemeris is about four hours.

**Figure 12.**
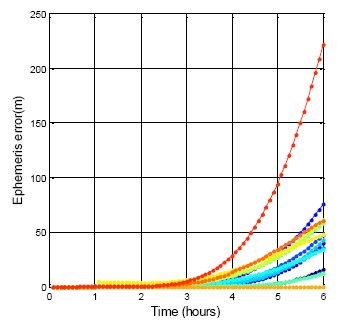
BDS DQA3: The same ephemeris used for 6 h.

The DQA3 result for the BDS is shown in Figure 12. Compare with the GPS satellites, all the BDS satellite position errors rise after three hours. The maximum difference after the six hours is 221.5 m. This error is greater than that of the GPS due to the shorter expired time of the ephemeris. The available period of the BDS ephemeris is defined in this paper as being three hours. This result is an hour faster than the GPS ephemeris. This also explains the why the BDS ephemeris must be updated every hour. It can be concluded from the ephemeris data comparison that the time and orbit prediction system of the BDS is not as mature as that of the GPS. The BDS ephemeris data needs an integrity system to validate a new incoming ephemeris. The user positioning result will be smoother after the new ephemeris has been tested first. On the other hand, the results show that the control of the BDS satellite (by the ground control station in China) still has room for improvement.

## 5. Data Quality Analysis 4 (DQA4): Satellite Position Difference between Almanac and Ephemeris

**Figure 13.**
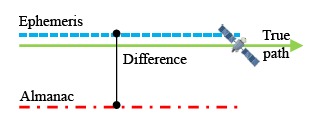
Schematic diagram of DQA4.

Unlike the ephemeris data, almanac data is not very precise for calculating satellite position. However, it can be used for several months. Almanac data is used when a GPS receiver is just turned on. It helps with regard to the time required for a GPS receiver to acquire satellite signals and navigation data and calculates a position solution. The approximate almanac position result is enough to decide which satellite needs to do the search. The almanac data helps a user to spend less time obtaining the first positioning result, which is also called time to first fix (TTFF). The almanac data includes all satellite orbital parameters. The total satellite orbit parameters can be received from one satellite. However, if the user wants to use the complete almanac data, it takes 12.5 min to download the complete navigational message. In this section, we verify the satellite position difference using the almanac and ephemeris, as shown in Figure 13.

**Figure 14.**
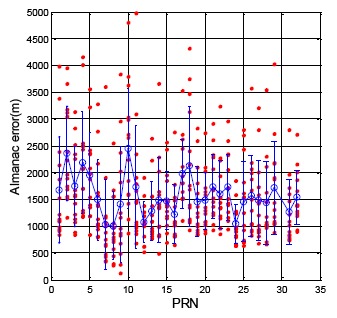
GPS DQA4: Satellite Position Difference between Almanac and Ephemeris.

The GPS almanac update rate is close to one day. Figure 14 shows the GPS satellite position difference between almanac and ephemeris, and GPS DQA4 statistical results are summarized in Table 5. The difference value was calculated when the new almanac received. The true satellite position is calculated from the ephemeris at the same time. Compare with the true satellite position, the mean value for all GPS satellite is 1537.8 m, the maximum is 4982.2 m and the standard deviation is 716.4 m.

**Figure 15.**
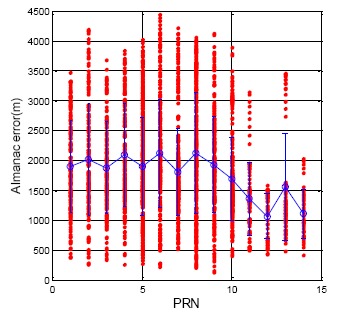
BDS DQA4: Satellite Position Difference between Almanac and Ephemeris.

The BDS almanac update rate is almost every hour. However, the almanac data is not synchronized for all BDS satellites. Sometimes the BDS MEO was broadcasting the old almanac. The time of almanac (TOA) is a reference time for users to calculate the satellite position. TOA usually is increased with the time during a one week period. The BDS TOA cannot provide a stable growing TOA. Sometimes, the user receives new almanac data, but the TOA is incorrectly tagged as occurring in the previous week. However, the entire BDS almanac still can provide adequate accuracy for the user. The results are presented in Figure 15. Table 6 shows the BDS DQA4 statistical results. The maximum position error for the BDS almanac data is 4448.9 m. The mean and standard deviation for the whole constellation is 1756.1 and 759.6 m, respectively. As a result, the BDS almanac data has almost the same accuracy as that of the GPS.

## 6. Data Quality Analyze 5 (DQA5): Applicable Almanac Period

**Figure 16.**
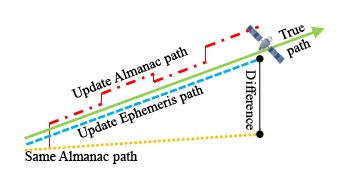
Schematic diagram of DQA5.

Although almanac data can be used for a longer time, it still has a useful life. In this part, this research applies the SIS almanac data for over a month in an attempt to find the applicable almanac period for both systems. The schematic diagram of the applicable almanac period test is presented in Figure 16. The true satellite position is calculated from the latest ephemeris data. Before verifying the almanac useful period, the detection mechanism should be established first. The purpose of the almanac is to use the rough positioning result to determine which satellite is in view. Moreover, the almanac also computes a rough estimate of the satellite Doppler shift. With the rough estimate Doppler shift, acquisition can be processed more quickly, and the information from the satellite can be decoded in a shorter period of time.

For a general GNSS signal, Equations (3) and (4) present the Doppler Effect for the signal. The *f* is the observed frequency; *f_0_* is the emitted frequency, and c is the speed of light. *v_r_* is the velocity of the receiver, which is positive if the receiver is moving towards the source. *v_s_* is the velocity of the source, which is positive if the source is moving away from the receiver. For Equation (4), if we redefine the *f_0_* as the observed frequency and *f* as the estimated frequency calculated from the almanac, then, the Δ*f* represents the error of the satellite Doppler shift estimate. For the same reason, if the *v_s_* and *v_r_* means the true satellite velocity and the velocity calculated from the almanac, the Δ*v* will be redefined as the velocity error between the truth and the value estimated from the almanac. The general case of the Doppler shift searching range is about 5000∼10,000 Hz, for which this research sets the Δ*f* as 7500 Hz. The GPS *f_0_* is set at 1575.42 MHz and BDS *f_0_* is 1561.098 MHz. With these assumptions, we can calculate the maximum velocity error for the GPS to be about 1427.203 m/s, and it is 1440.296 m/s for the BDS:

(3) $$f=\left(\frac{c+v_{r}}{c+v_{s}}\right)f_{0}$$

(4) $$\Delta f=\frac{\Delta v}{c}f_{0}\;\text{where},\Delta f=f-f_{0},\Delta v=v_{r}-v_{s}$$

(5) $$\Delta v=\frac{\Delta f}{f_{0}}c\;\text{where},\Delta f=f-f_{0},\Delta v=v_{r}-v_{s}$$

**Figure 17.**
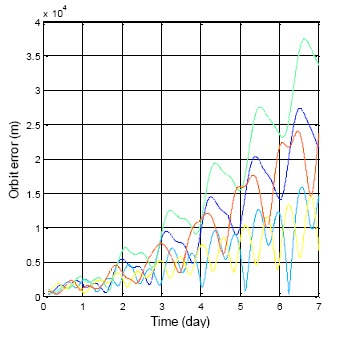
BDS GEO satellite position error by using the same almanac for one week.

**Figure 18.**
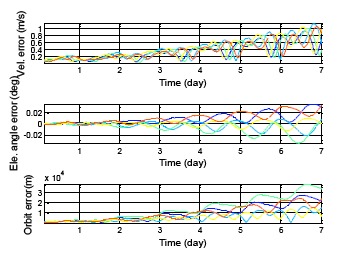
BDS GEO almanac using period test for one week.

**Figure 19.**
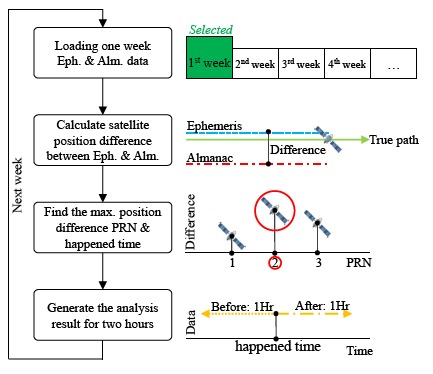
BDS DQA1: The flow chart for generating the applicable almanac period.

Figure 17 presents the satellite positioning error for the same almanac use for one week. The true position is using the satellite position calculated from the ephemeris. This result requires a continuous true position. The BDS GEO satellites have been selected in this analysis due to the fact that users in Taiwan can continuously receive these satellite signals. The different colors represent the different BDS GEO satellites in Figure 17. The results show that all positioning errors grow with time, and the maximum position error after one week is more than 35,000 m. In this section, the satellite position error is not the only indicator. The elevation angle error and velocity error for each satellite also become analysis items. Figure 18 shows the all analysis indicators in the DQA5. The top figure demonstrates the velocity error, which relates to using the almanac to estimate the Doppler shift. The middle figure presents the elevation angle error which relates to using the almanac to select the satellites in view. The bottom plot of Figure 19 is the original analysis, which shows the almanac positioning error. The velocity and elevation angle still have enough accuracy after one week. The receiver can use the almanac of an age less than one week to shorten the TTFF.

For the other constellation satellites, the user cannot continuously receive the ephemeris data due to the fact that the satellite will move outside the line of sight. In the following analysis, the maximum positioning error when the satellite ephemeris can be used during one week is calculated. As shown in Figure 19, first, one week of ephemeris and almanac data is used to calculate the satellite positioning difference for each satellite. After that, each positioning difference is compared to select the maximum satellite PRN number and record the occurrence time for that satellite. This selected satellite represents the maximum almanac error during this week. Finally, the maximum satellite information *i.e.*, the velocity error, elevation angle error and the positioning error for two hours is calculated. These two hour analysis results are composed of one hour of data before the maximum positioning error and one hour of data after the maximum positioning error. After generating all of the results, it is necessary to go back to the first step and calculate the data for the following week.

**Figure 20.**
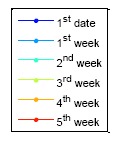
BDS DQA1: The legend of the Figure 21 to 24.

**Figure 21.**
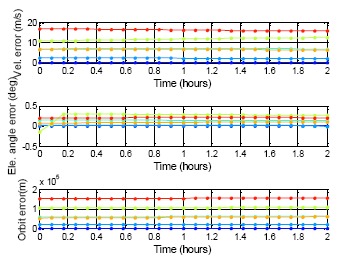
GPS almanac using period test for five weeks.

The following results presents the almanac use period for a five-week estimation. Figure 20 shows the color information for the following results: Figure 21 is the GPS five week estimation, and the statistical results are shown in Table 7. The velocity error for the maximum satellite at the fifth week is under 20 m/s. It is still far away from the threshold, which is 1427.203 m/s according to the calculation discussed in the previous paragraph. The elevation angle error for the fifth week is not more than 0.5 degrees, which is also accurate enough for satellite selection. Finally, the positioning error for the fifth week is more than 150,000 m. The result show the GPS almanac could be used for over five weeks.

**Figure 22.**
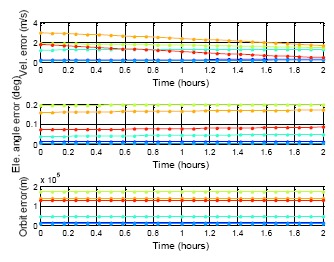
BDS DQA1: BDS GEO almanac using period test for five weeks.

**Figure 23.**
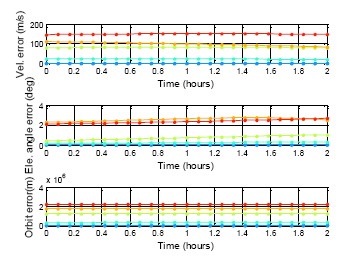
BDS DQA1: BDS IGSO almanac using period test for five weeks.

**Figure 24.**
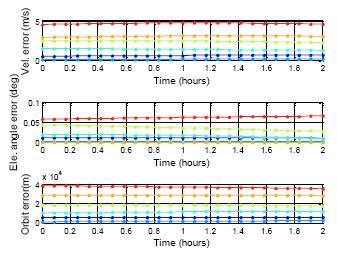
BDS MEO almanac using period test for five weeks.

Figures 22 to 24 show the estimation results for the BDS GEO, IGSO, and MEO, respectively. The statistical results for these analyses are also demonstrated in Table 7. For each analysis item, the BDS GEO and MEO satellites exhibited better estimation than the GPS satellites. However, the BDS IGSO satellites showed excessive error on each analysis item starting from the third week. The velocity grew to almost 150 m/s at the fifth week, and the final positioning error was more than 2,000,000 m. Moreover, the most serious error occurred on the elevation angle. The elevation angle error in the third week was over 1 degree. This result would influence the selection of the satellite-in-view. The almanac failed to estimate the satellite movement due to the fact that the information had expired, or this satellite was subjected to some external influences and departed from the original planned orbit. If one satellite changed its direction, the position prediction required new information. Otherwise the prediction error would grow over time. As a result, the control of the IGSO satellite still needs to be improved. Until this problem is solved, this research suggested that users update the BDS almanac information every time the user receives the BDS signal.

## 7. Conclusions and Future Work

This study collected actual SIS GPS and BDS data to present a detailed comparative study of GPS and BDS navigation data. The GPS ephemeris and almanac data were treated as a baseline by which to evaluate the BDS ephemeris and almanac data. The five DQAs proposed in this paper successfully captured the anomalies resulting from the use of BDS navigation data to calculate the satellite information. Specifically, the DQA1 detected that BDS IGSO and MEO have a larger position difference than that of the GPS when ephemeris updates. BDS users could use the DQA1 mechanism to test a new ephemeris and remove the irregular ephemeris data in order to have a stable satellite position solution. Compared to the GPS, the DQA2 showed that all of the BDS satellites have larger clock differences when the ephemeris updates. That is, the BDS ephemeris clock corrections accuracy is insufficient. BDS users are recommended to have an additional estimation scheme for the BDS satellite clock correction. The DQA3 estimates the applicable period of the ephemeris data, and for the same working accuracy level, the GPS ephemeris could be used for 4 h, and the BDS ephemeris could be used for 3 h. According to these results, if one BDS satellite does not receive a new ephemeris update, then a BDS user should remove the old ephemeris data after 3 h. The DQA4 verified that the satellite position differences between the almanac and ephemeris for both systems are of almost the same quality. Finally, the DQA5 validated the applicable almanac data periods for both systems. As shown in the results, the GPS almanac could be applied for over 5 weeks. On the other hand, the BDS IGSO elevation angle estimates failed after the third week.

In conclusion, the BDS can provide positioning service as good as that of the GPS most of the time. However, sometimes the BDS will have irregular ephemeris and almanac data information, and BDS users will not be able to discover these anomalies using a conventional GNSS receiver. Importantly, the probability and magnitude of unusual BDS ephemeris and almanac data events are larger than those of the GPS. Thus, if one would like to integrate the GPS and BDS for navigation and guidance, then additional navigation data quality analyses as proposed in this paper are needed.

## Figures and Tables

**Table 1.** Comparison of satellite constellation.

	**GPS**	**BDS**		
**Orbit**	MEO	GEO	IGSO	MEO
**Orbit Radius**	20,200	35,786	35,786	21,528
**Coordinate**	WGS84	CGCS2000		
**Time**	GPST	BDT		
**Time Start**	January 6th, 1980	January 1st, 2006		

**Table 2.** The state of the SIS ephemeris data.

**Flag**	**Receive State**
**0**	New data incoming
**1**	Data update
**2**	Expired/Unhealthy data

**Table 3.** BDS DQA1 statistical results.

GEO					
PRN	1	2	3	4	5
Mean	0.37	0.32	0.35	0.45	0.48
std	0.25	0.19	0.25	0.31	0.29

IGSO					

PRN	6	7	8	9	10
Mean	0.33	0.34	0.31	0.24	0.86
std	0.22	0.22	0.24	0.17	2.99

MEO					

PRN	11	12	13	14	X
Mean	5.37	6.83	0.44	0.46	X
std	8.77	12.95	0.66	0.67	X

**Table 4.** Statistical results for BDS DQA2.

GEO					
PRN	1	2	3	4	5
Mean	−0.02	−0.03	0.00	0.05	0.03
std	0.24	0.38	0.11	0.11	0.19

IGSO					

PRN	6	7	8	9	10
Mean	−0.01	−0.05	−0.03	0.04	0.00
std	0.37	0.41	0.27	0.27	0.26

MEO					

PRN	11	12	13	14	X
Mean	0.17	0.20	−0.03	0.06	X
std	0.59	0.41	1.30	0.64	X

**Table 5.** GPS DQA4 statistical results.

**PRN**	**1**	**2**	**3**	**4**	**5**	**6**	**7**	**8**
**Mean**	1680.7	2364.1	1748.9	2189.5	1955.2	1497.1	1032.5	1007.1
**std**	980.3	877.3	743.7	953.7	785.1	753.6	849.9	631.5
**PRN**	**9**	**10**	**11**	**12**	**13**	**14**	**15**	**16**
**Mean**	1405.5	2451.0	1728.8	1079.2	1283.3	1487.9	1476.6	1222.7
**std**	1076.1	1130.4	1145.4	380.1	557.4	791.3	548.9	549.4
**PRN**	**17**	**18**	**19**	**20**	**21**	**22**	**23**	**24**
**Mean**	1984.2	2121.2	1448.7	1478.6	1738.5	1597.6	1730.8	1040.6
**std**	644.0	1120.9	657.7	529.9	611.0	633.3	619.9	363.2
**PRN**	**25**	**26**	**27**	**28**	**29**	**30**	**31**	**32**
**Mean**	1459.3	1566.9	1477.5	1442.5	1711.4	X	1265.0	1535.8
**std**	750.4	748.6	758.1	771.9	869.6	X	594.5	498.3

**Table 6.** BDS DQA4 statistical results.

**GEO**					
PRN	1	2	3	4	5
Mean	1904.1	2023.6	1879.2	2099.9	1897.7
std	774.4	940.1	765.9	866.1	828.0

**IGSO**					

PRN	6	7	8	9	10
Mean	2124.6	1811.1	2126.3	1933.3	1689.7
std	906.3	729.0	1017.8	812.9	692.5

**MEO**					

PRN	11	12	13	14	X
Mean	1361.4	1065.3	1560.9	1108.1	X
std	611.7	380.0	893.4	415.9	X

**Table 7.** DQA5 statistic result.

**GPS**					

**MAX**	**PRN**	**Vel.**	**E.A.**	**Orbit**
1st date	3	0.35	0.003	2079.03
1st week	26	2.63	0.052	23,328.54
2nd week	26	6.80	0.125	60,979.13
3rd week	26	12.48	0.302	107,782.35
4th week	32	6.80	0.103	60,190.15
5th week	26	18.71	0.427	161,347.99

**BDS GEO**					

**MAX**	**PRN**	**Vel.**	**E.A.**	**Orbit**

1st date	5	0.20	0.003	4017.31
1st week	3	0.37	0.030	52,818.54
2nd week	3	1.70	0.115	134,449.08
3rd week	1	2.01	0.327	293,921.55
4th week	1	3.21	0.342	302,857.73
5th week	1	2.75	0.292	328,923.79

**BDS IGSO**					

**MAX**	**PRN**	**Vel.**	**E.A.**	**Orbit**

1st date	10	0.23	0.003	3388.02
1st week	7	4.90	0.013	11,788,168.81
2nd week	10	21.86	0.297	327,414.07
3rd week	10	80.69	1.015	1,201,839.57
4th week	10	108.70	2.693	1,697,328.75
5th week	10	147.73	2.633	2,188,705.08

**BDS MEO**					

**MAX**	**PRN**	**Vel.**	**E.A.**	**Orbit**

1st date	14	0.10	0.002	800.21
1st week	12	1.84	0.033	14,524.20
2nd week	12	2.58	0.044	19,293.95
3rd week	12	5.25	0.071	39,728.10
4th week	11	5.31	−0.077	38,446.30
5th week	12	10.50	0.189	81,091.40
